# Respiration Interacts With Photosynthesis Through the Acceptor Side of Photosystem I, Reflected in the Dark-to-Light Induction Kinetics of Chlorophyll Fluorescence in the Cyanobacterium *Synechocystis* sp. PCC 6803

**DOI:** 10.3389/fpls.2021.717968

**Published:** 2021-07-28

**Authors:** Takako Ogawa, Kenta Suzuki, Kintake Sonoike

**Affiliations:** Faculty of Education and Integrated Arts and Sciences, Waseda University, Tokyo, Japan

**Keywords:** photosynthesis, respiration, chlorophyll fluorescence, cyanobacteria, NADPH, redox regulation

## Abstract

In cyanobacteria, the photosynthetic prokaryotes, direct interaction between photosynthesis and respiration exists at plastoquinone (PQ) pool, which is shared by the two electron transport chains. Another possible point of intersection of the two electron transport chains is NADPH, which is the major electron donor to the respiratory chain as well as the final product of the photosynthetic chain. Here, we showed that the redox state of NADPH in the dark affected chlorophyll fluorescence induction in the cyanobacterium *Synechocystis* sp. PCC 6803 in a quantitative manner. Accumulation of the reduced NADPH in the dark due to the defect in type 1 NAD(P)H dehydrogenase complex in the respiratory chain resulted in the faster rise to the peak in the dark-to-light induction of chlorophyll fluorescence, while depletion of NADPH due to the defect in pentose phosphate pathway resulted in the delayed appearance of the initial peak in the induction kinetics. There was a strong correlation between the dark level of NADPH determined by its fluorescence and the peak position of the induction kinetics of chlorophyll fluorescence. These results indicate that photosynthesis interacts with respiration through NADPH, which enable us to monitor the redox condition of the acceptor side of photosystem I by simple measurements of chlorophyll fluorescence induction in cyanobacteria.

## Introduction

Metabolic pathways are often separately described in simple schematic diagrams in textbooks. However, interactions between different metabolic pathways through common metabolites are universally observed. Although these interactions may not be so conspicuous in eukaryote, much stronger interaction is observed in prokaryotes, which have no organelle to compartment metabolic pathways. In cyanobacteria, photosynthetic prokaryotes, photosynthesis can be affected by a wide range of other metabolic pathways. To examine such metabolic interaction, the dark-to-light induction curve of chlorophyll fluorescence, so-called the Kautsky curve (Kautsky and Hirsch, 1931; Govindjee, 1995), is useful. The analysis of the “Fluorome” database,^1^ the database of the Kautsky curve of mutants of 750 genes created by random mutagenesis using transposon (Ozaki et al., 2007), revealed that the disruption of the two-thirds of the genes examined affected the Kautsky curve in one way or others (Ozaki and Sonoike, 2009). Apparently, the disruption of not only “photosynthesis-related” genes but also many genes that are not directly related to photosynthesis affects the condition of photosynthesis.

Even among prokaryotes, the interaction between photosynthesis and respiration in cyanobacteria is especially conspicuous, because they share several components of electron transport chain, such as plastoquinone (PQ), cytochrome *b*_6_/*f* complex, and plastocyanin (e.g., Aoki and Katoh, 1982; Peschek and Schmetterer, 1982). Actually, when searched in Fluorome database mentioned above, the most drastic change in the Kautsky curve is observed in the gene-disrupted mutant of the *ndhF1* (Δ*ndhF1*), which encodes a subunit of type 1 NAD(P)H dehydrogenase (NDH-1) complex in the respiratory electron transport chain (Mi et al., 1992; Battchikova et al., 2011). The disruption of the *ndhF1* gene brought about very high peak and the faster rise in the Kautsky curve. The cause of the high peak observed in Δ*ndhF1* was ascribed to the oxidation of the PQ pool in the dark due to poor electron supply from NDH-1 complex in the respiratory chain, since the height of the peak in Δ*ndhF1* decreased to the wild-type level by the addition of KCN, which is an inhibitor of terminal oxidase, and thus leading to the reduction of the PQ pool (Ogawa et al., 2013). The result suggests that respiration drastically affects condition of photosynthesis through the interaction at the PQ pool not only in the dark but also under illumination, even though the rate of respiratory electron transport is approximately one order smaller than the maximum rate of photosynthetic electron transport. Interestingly, faster induction of chlorophyll fluorescence observed in Δ*ndhF1* was hardly affected by the addition of KCN (Ogawa et al., 2013). These results suggest that, while the peak height of the Kautsky curve reflects the redox state of the PQ pool in the dark before the measurements, the rate of fluorescence induction toward the peak may mainly reflect the redox state of another component in the electron transport chain.

As a candidate for such component, we assume that nicotinamide adenine dinucleotide phosphate (NADP) is the most plausible one, since reduced NADP (NADPH) is the major electron donor to the respiratory electron transport chain in cyanobacteria (Biggins, 1969) while it is the final product of the linear electron transport of photosynthesis. Actually, reduced NADPH was reported to be accumulated in the dark in the NDH-1 defective mutant of *Synechocystis* sp. PCC 6803 because of the slow oxidation of NADPH by NDH-1 (Mi et al., 2000). The resulting deficiency of NADP^+^, the final electron acceptor of photosynthetic electron transport, may be the cause of the faster induction of chlorophyll fluorescence observed in Δ*ndhF1*.

In this report, we compared two strains of the cyanobacterium *Synechocystis* sp. PCC 6803, in which the redox states of NADPH are different from the wild-type strain and from each other while that of the PQ pool in the dark is similar. Here, we demonstrate that the rate of fluorescence induction toward peak level is mainly dependent on the redox state of NADPH after dark-acclimation. Apparently, condition of respiration affects photosynthesis not only through the PQ pool but also through the acceptor side of photosystem I (PSI), reflected in the dark-to-light induction curve of chlorophyll fluorescence in *Synechocystis* sp. PCC 6803. Furthermore, it was shown that time to reach a peak of induction of chlorophyll fluorescence can be used as an index of the redox state of NADPH.

## Materials and Methods

### Strains and Growth Conditions

The gene-disrupted mutants of *ndhF1* and *gnd*, which were originally used to determine the fluorescence induction kinetics for the Fluorome database (see footnote 1), had been constructed by transposon mutagenesis of *Synechocystis* sp. PCC 6803 and conferred resistance to chloramphenicol (Ozaki et al., 2007). The wild-type strain and the gene-disrupted mutants were grown at 30°C in BG11 medium (Allen, 1968), buffered with 20 mM TES-KOH (pH 8.0), and bubbled with air for 24 h under continuous illumination using fluorescent lamps from two sides. Photon flux density of the growth light was 120 μmol m^−2^ s^−1^, determined by a spherical micro-sensor (US-SQS/L, Walz) with a light meter (LI-250, LI-COR Biosciences). Chloramphenicol at 25 μg ml^−1^ was added to the culture medium for the growth of the gene-disrupted mutants.

### Chlorophyll Fluorescence Emission Spectra Determined at 77 K

Chlorophyll fluorescence emission spectra were measured at 77 K with a fluorescence spectrometer (FP-8500, JASCO) with a low temperature attachment (PU-830, JASCO) as described in Ogawa et al. (2013). Cell suspensions were adjusted to a chlorophyll concentration of 2 μg ml^−1^. Prior to the measurements, the cells were incubated for 10 min at room temperature either in the dark, or in the dark in the presence of 0.2 mM KCN, or under growth light, or under high light at 550 μmol m^−2^ s^−1^ in the presence of 10 μM 3-(3,4-dichlorophenyl)-1,1-dimethylurea (DCMU).

### Measurements of Kautsky Transient

Chlorophyll fluorescence induction kinetics under illumination at 200 μmol m^−2^ s^−1^ was measured by a fluorescence CCD camera (FluorCam 800MF, Photon System Instruments). Five ml of cell cultures with an optical density of 0.5 at 750 nm was put in a plastic petri dish with 3.5 cm diameter, and then served for the measurements. The optical density of the cell cultures was measured by a spectrophotometer (V-650, JASCO). Cells were kept in the dark for 15 min prior to the measurement. Time to reach the initial peak of the Kautsky curve of chlorophyll fluorescence was determined by fitting the curve to cubic function to calculate the time point giving the maximum value.

### Simultaneous Measurements of NADPH Fluorescence and Chlorophyll Fluorescence

Simultaneous measurements of NADPH fluorescence and chlorophyll fluorescence were performed by a fluorometer (Dual-PAM-100, Walz) equipped with NADPH/9-AA modules, which consisted of the following emitter and detector units. A unit (DUAL-ENADPH, Walz) emitted UV-A (365 nm) and red (620 nm) measuring light to excite NADPH and chlorophyll, respectively. NADPH fluorescence was detected by another unit (DUAL-DNADPH, Walz) of a blue-sensitive photomultiplier with filters transmitting 420–550 nm light. Chlorophyll fluorescence was detected by a unit (DUAL-DPD, Walz) protected with an RG665 filter, which blocks short-wavelength light and only passes chlorophyll fluorescence. A saturating pulse for 800 ms was provided by a chip-on-board LED (635 nm) of the DUAL-ENADPH unit. The chlorophyll concentration of the cell suspension, determined as described in Grimme and Boardman (1972), was adjusted to 10 μg ml^−1^. Prior to the measurements, cells were illuminated by red light at 16 μmol m^−2^ s^−1^ for 1 min followed by dark-acclimation for 15 min. Levels of chlorophyll fluorescence (Ft) were normalized with the average of levels during 200 ms before the onset of saturating pulse (Fo) and the peak level under illumination with saturating pulse (Fp) as 0 and 1, respectively. To quantify the time to reach the peak level, we defined an index “T_peak_” as time to reach 93% of the peak level. Since the fluorescence signal contains significant noise, the Ft usually crossed the line representing (Ft − Fo)/(Fp − Fo) = 0.93 several times. So, we first determined the time range between the point Ft first crosses the line and the point Ft last crosses the line. Then, approximation straight line was fitted to the Ft in that time range, and interception point between the straight line and (Ft − Fo)/(Fp − Fo) = 0.93 was defined as “T_peak_.” This ratio (93%) was determined to make the uncertainty (ambiguity of the time point due to the noise) minimum relative to a range of the parameter.

### Estimation of the Available Oxidizable Equivalents of the PQ Pool per Q_A_ in the Dark

The dark-to-light induction curve of chlorophyll fluorescence upon saturating pulse for 800 ms was measured by using a fluorometer (Dual-PAM-100, Walz) equipped with NADPH/9-AA modules as described above, but measuring light for NADPH fluorescence was turned off. Cells were treated with 10 μM DCMU or 20 μM 2,5-dibromo-3-methyl-6-isopropyl-*p*-benzoquinone (DBMIB) in the presence of 5 mM ascorbate, followed by dark-acclimation for 15 min prior to the measurements. Measuring light for chlorophyll fluorescence was set to minimum to avoid the reduction of Q_A_ in the presence of DCMU. The relative area above the induction curve was calculated as the sum of (Fp − Ft)/(Fp − Fo) during the first 500 ms of the measurement in the presence of either DCMU (R_DCMU_) or DBMIB (R_DBMIB_). The number of available oxidized equivalents in the PQ pool (N) was estimated as N=(R_DBMIB_-R_DCMU_)/R_DBMIB_ (Berry et al., 2002).

## Results

**Figure 1:**
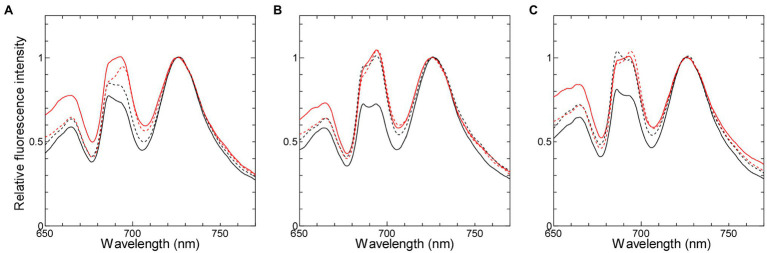
77 K chlorophyll fluorescence emission spectra with phycocyanin excitation at 625 nm in the wild-type strain **(A)**, Δ*ndhF1* **(B)**, and Δ*gnd* **(C)**. Black solid line, dark-acclimated cells in the presence of 0.2 mM KCN; black dashed line, dark-adapted cells without any addition; red dashed line, growth light-acclimated cells; and red solid line, high light-acclimated cells in the presence of 10 μM DCMU. Each fluorescence spectrum was normalized at the PSI fluorescence peak at 725 nm. The averaged spectra of three independent cultures are presented except for the spectrum of the growth light-acclimated Δ*ndhF1* cells (average of two independent cultures).

We used two cyanobacterial strains, in which the redox states of NADPH are different but those of the PQ pool are similar after dark-acclimation, in order to verify the effect of NADPH on chlorophyll fluorescence. One of the two strains is Δ*ndhF1*, in which the PQ pool is oxidized (Ogawa et al., 2013) while NADPH is reduced in the dark (Mi et al., 2000; Tanaka et al., 2021). The other is the strain with disrupted *gnd* gene (Δ*gnd*), which encodes 6-phosphogluconate dehydrogenase (Kaneko et al., 1996) in oxidative pentose phosphate (OPP) pathway, the major pathway producing NADPH in the dark (Cheung and Gibbs, 1966; Wolk, 1973). In Δ*gnd*, both NADPH and the PQ pool should be oxidized during dark-acclimation, because NADP^+^ cannot be reduced without OPP pathway and the PQ pool cannot be reduced as well in the absence of the electron donor to the respiratory chain. The oxidized PQ pool in the dark-acclimated Δ*gnd* cells was confirmed by 77 K chlorophyll fluorescence emission spectra with phycobilisome excitation (Figure 1), which reflected state transition depending on mobile phycobilisome regulated by the redox state of the PQ pool (Mullineaux and Allen, 1986; Mullineaux et al., 1997). When cells were illuminated in the presence of DCMU which inhibits electron transport from Q_A_ to Q_B_ in photosystem II (PSII), the PQ pool was fully oxidized to lock the cells in State 1, reflected in higher ratio of PSII fluorescence around 685–695 nm to PSI fluorescence around 725 nm (Figures 1A–C, red solid lines). On the other hand, when cells were dark-acclimated in the presence of KCN, an inhibitor of terminal oxidase in the respiratory chain, the PQ pool was fully reduced and State 2 was induced, reflected in lower ratio of PSII/PSI fluorescence (black solid lines). The spectra of cells simply dark-acclimated with no KCN (black dashed line) were similar to those of the cells with the fully reduced PQ in the wild-type strain (Figure 1A), while they were similar to those of the cells with the fully oxidized PQ in Δ*gnd* (Figure 1C) as well as in Δ*ndhF1* (Figure 1B; see also Ogawa et al., 2013). These results indicate that the PQ pool is fully oxidized during dark-acclimation in the two gene-disrupted mutants in contrast to the case of the wild-type strain.

It is interesting to note that the peak height around 695 nm relative to that around 685 nm (F_695_/F_685_) varied in different experimental conditions (Figure 1). Similar variation in the F_695_/F_685_ ratio has been also observed in *Synechococcus elongatus* PCC 6301 (Mullineaux and Allen, 1990) or even in glaucophyte (Misumi and Sonoike, 2017), suggesting that the change is widely observed in phycobilisome-containing organisms. The origin of the fluorescence peak around 685 nm or that around 695 nm was first assigned to the PSII core subunits, CP43 (PsbC) or CP47 (PsbD), respectively (Yamagishi and Katoh, 1983). Furthermore, terminal emitter of phycobilisome also contributes to the 685 nm fluorescence (Bruce et al., 1985). Since the F_695_/F_685_ ratio is higher under State 1 condition than under State 2 condition in the wild-type strain and Δ*ndhF1*, we assume that the most plausible explanation is the enhanced emission from the terminal emitter in State 2. It is well known that some of phycobilisomes are detached from PSII and transfer its absorbed light energy to PSI under State 2 condition. In the remaining phycobilisomes, energy transfer from phycobilisomes to PSII may become somewhat inefficient under State 2 condition, leading to the increased fluorescence around 685 nm. In Δ*gnd*, however, some additional mechanism must be considered, since dark-acclimated cells showed high 686 nm peak (Figure 1C, black dashed line), although the cells must be in State 1.

The oxidized PQ pool in the dark-acclimated cells of the two gene-disrupted mutants was confirmed by the measurement of the dark-to-light induction curve of chlorophyll fluorescence at room temperature (Supplementary Figure 1). Since the area above the induction curve is proportional to the number of electrons passing through PSII, the ratio of the area determined with DBMIB to that with DCMU can be used for the measure of the available oxidized equivalents of the PQ pool per Q_A_ in the dark-acclimated cells. In the wild-type strain, the area determined with DBMIB was almost identical to that with DCMU (compare green line and orange line in Supplementary Figure 1A). Thus, the number of available oxidized equivalents of the PQ pool per Q_A_ (N) is very small (−0.05 ± 0.05), suggesting that the PQ pool was almost fully reduced in the dark-acclimated wild-type cells. Much larger N was obtained for the two gene-disrupted mutants (4.21 ± 1.29 in Δ*ndhF1* and 9.62 ± 2.27 in Δ*gnd*) compared with the wild-type strain. These results indicate that the PQ pool was oxidized during dark-acclimation of the two gene-disrupted mutants, supporting the results of the chlorophyll fluorescence emission spectra determined at 77 K (Figure 1).

**Figure 2:**
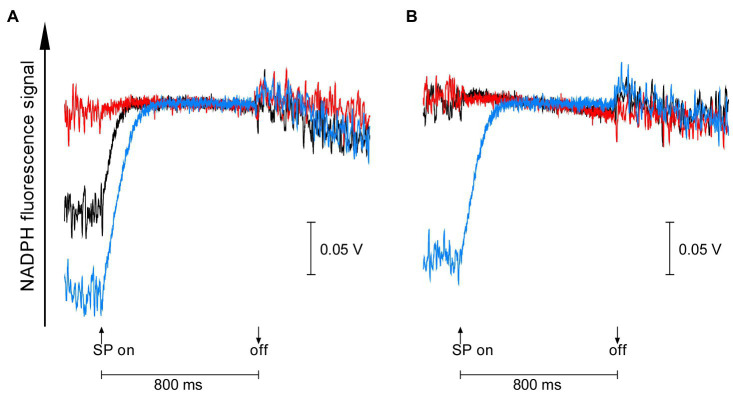
The dark-to-light induction kinetics of NADPH fluorescence upon saturating pulse (SP) of 800 ms in the absence **(A)** or presence **(B)** of glucose. Black line, the wild-type strain; red line, Δ*ndhF1*; and blue line, Δ*gnd*. Prior to measurements, cells were dark-acclimated for 15 min. The average of kinetics of three independent cultures in each strain is presented.

On the other hand, the redox state of NADPH in the dark-acclimated cells of the two gene-disrupted mutants was quite different from each other. NADPH fluorescence of dark-acclimated cells increased upon 800 ms pulse of saturating light in the wild-type strain (black line in Figure 2), reflecting the reduction of NADP^+^ accumulated in the dark. Similar but much larger increase in NADPH fluorescence was observed in Δ*gnd*, indicating that more NADP^+^ was accumulated during dark-acclimation (blue line in Figure 2A). In the case of Δ*ndhF1*, however, no increase in NADPH fluorescence was observed so that NADPH had been already fully reduced in the dark. These results suggest that the acceptor side of PSI must be more oxidized in the dark-acclimated Δ*gnd* cells compared with the wild-type cells, while NADPH is fully reduced in the dark-acclimated Δ*ndhF1* cells. The redox state of NADPH can be modified by the addition of glucose, a substrate for the OPP pathway. Upon addition of glucose, the light-induced NADPH fluorescence rise in the wild-type strain was diminished as observed in Δ*ndhF1* (black line and red line in Figure 2B). This result indicates that NADPH is almost fully reduced in the dark-acclimated wild-type cells in the presence of glucose. In the case of Δ*gnd*, the addition of glucose had only a marginal effect on the light-induced NADPH fluorescence rise (blue line in Figure 2B).

**Figure 3:**
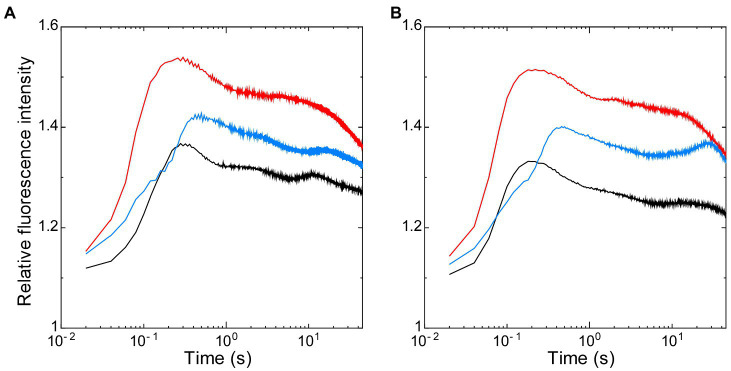
The dark-to-light induction kinetics of chlorophyll fluorescence measured in the absence **(A)** or presence of glucose **(B)**. Black line, the wild-type strain; red line, Δ*ndhF1*; and blue line, Δ*gnd*. Prior to measurements, cells were dark-acclimated for 15 min. The intensity of chlorophyll fluorescence was normalized at the onset of actinic illumination. The average of the kinetics of three independent cultures in each strain is presented.

Thus, using these two strains that have the opposite redox state in NADPH with the similarly oxidized PQ pool after dark-acclimation, we first analyzed the effects of the redox state of NADPH on the dark-to-light induction kinetics of chlorophyll fluorescence, so-called Kautsky curve (Figure 3). The initial peak of the Kautsky curve appeared earlier in Δ*ndhF1* (202 ± 31 ms after onset of illumination) when compared to the wild-type strain (261 ± 63 ms) (compare red line with black line in Figure 3A) as we have previously reported (Ogawa et al., 2013). On the other hand, the appearance of the peak is delayed in Δ*gnd* (447 ± 26 ms; blue line in Figure 3A). In the presence of glucose, the initial peak of the fluorescence induction appeared significantly earlier in the wild-type strain (182 ± 11 ms, approximately 70% of the time with no addition; black line in Figure 3B), while smaller effect of glucose was observed in Δ*ndhF1* (166 ± 1 ms, approximately 82% of the time with no addition) and Δ*gnd* (434 ± 37 ms, approximately 97% of the time with no addition; red or blue line in Figure 3B, respectively).

**Figure 4:**
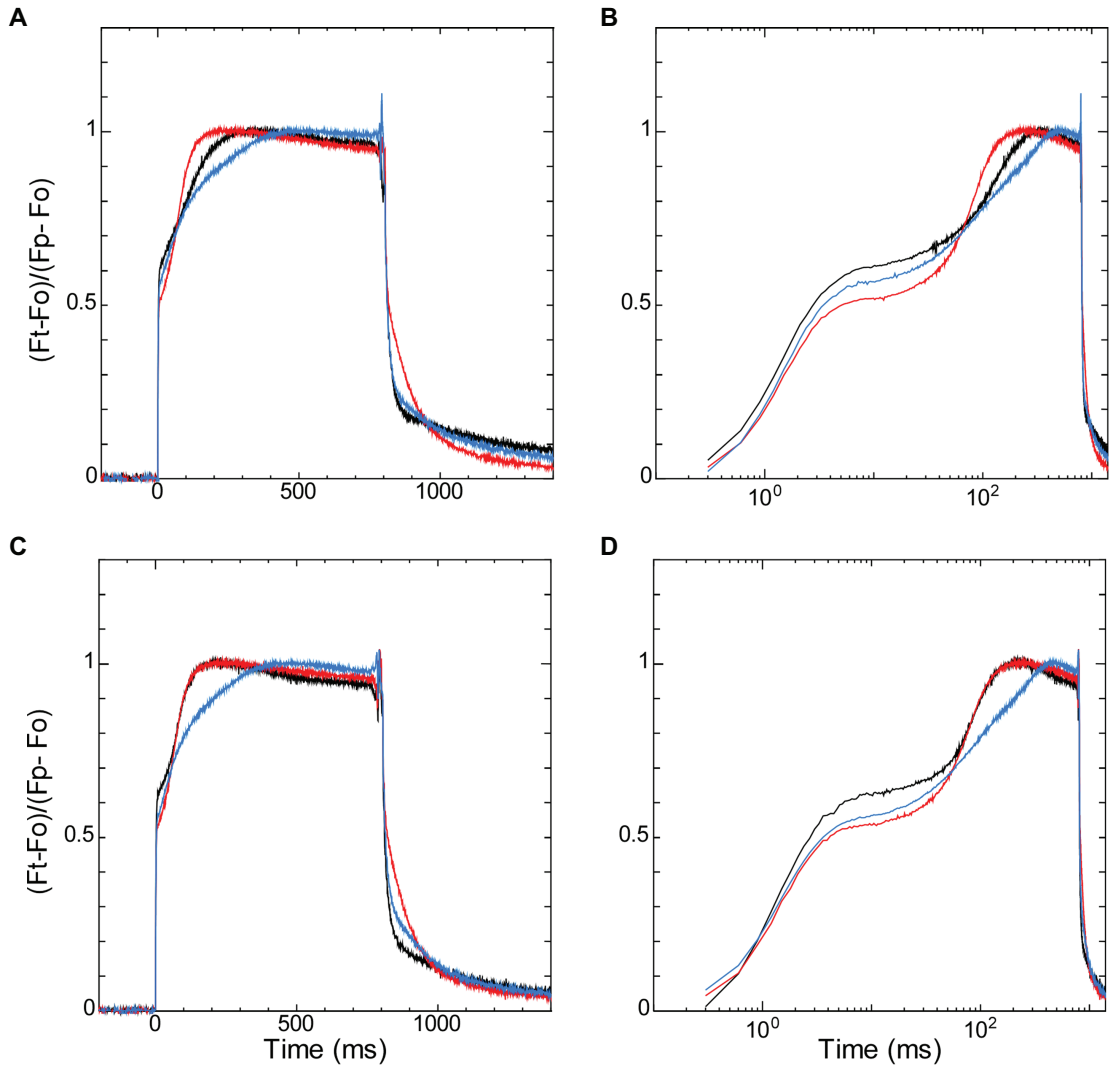
The dark-to-light induction kinetics of chlorophyll fluorescence upon saturating pulse of 800 ms measured in parallel with NADPH fluorescence (Figure 2) in the absence **(A,B)** or presence **(C,D)** of glucose. Fluorescence data on the left panels **(A,C)** are plotted against linear scale and the right panels **(B,D)** are plotted against logarithmic scale. Black line, the wild-type strain; red line, Δ*ndhF1*; and blue line, Δ*gnd*. Fluorescence signals are normalized by Fo and Fp as 0 and 1, respectively (see Materials and Methods). The average of the kinetics of three independent cultures in each strain is presented.

The redox states of NADPH and the PQ pool in the dark-acclimated cells also affected fast induction of chlorophyll fluorescence by saturating pulse, so-called “OJIP” transient. It is known that chlorophyll fluorescence under high irradiance rises from the dark level (named “O”) to the peak (named “P”) with the two inflection points (named “J” and “I”; Strasser and Govindjee, 1991; Govindjee, 1995; Strasser et al., 2004; Papageorgiou et al., 2007). J-step and I-step are generally observed at about 2 ms and 30 ms after the onset of illumination, respectively (Strasser et al., 2004; Stirbet and Govindjee, 2011). We compared the induction curves of the wild-type strain with those of Δ*ndhF1* and Δ*gnd* by normalizing the time-dependent level (Ft) with regarding P-level (Fp) and O-level (Fo) as 1 and 0, respectively (Figure 4). The initial rise from O-step to the first inflection point (i.e., J-step) was observed in the order of 10 ms from the start of illumination, and the J-level was high in the wild-type strain and low in the two gene-disrupted mutants (Figure 4A in linear scale and Figure 4B in log scale). Interestingly, three curves crossed each other near the second inflection point (i.e., I-step) at around 70 ms from the start of illumination, and after that crossing, the fluorescence level is high in Δ*ndhF1* and low in Δ*gnd*, with the wide-type strain in the middle.

The addition of glucose did not affect the initial induction kinetics of fluorescence until the crossing point around I-step at 70 ms (Figures 4C,D). After the crossing point, however, the effect of glucose was observed in the wild-type strain: The induction became faster in the wild-type strain to the level of that in Δ*ndhF1*. Apparently, initial rise of fluorescence during the first 70 ms (corresponding to O-J-I phase) and subsequent induction to the peak level (corresponding to I-P phase) reflects different steps of photosynthesis (Strasser et al., 1995, 2004; Stirbet and Govindjee, 2011). The rise to J-level is fast in wild-type strain and slow in the two disruptants, presumably reflecting the redox state of PQ in the dark-acclimated cells. On the other hand, the subsequent induction to the P-level should reflect the redox state of NADPH in the dark-acclimated cells, considering the opposite redox state of NADPH in the two gene-disrupted mutants. This assumption was consistent with the observation in the Kautsky curve that the induction is faster in Δ*ndhF1* while it is slower in Δ*gnd* (Figure 3A).

**Figure 5:**
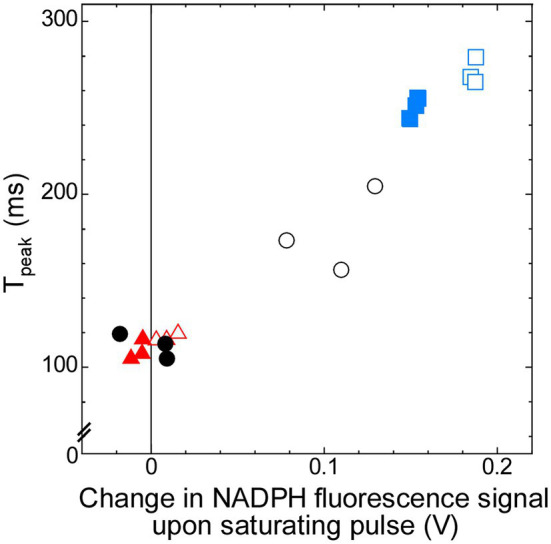
Correlation (*R*^2^ = 0.952) between T_peak_, which represents time to reach 93% of P-level of chlorophyll fluorescence (calculated from the data in Figure 4), and change in NADPH fluorescence signal upon saturating pulse (calculated from the data in Figure 2). Open symbols, cells measured in the absence of glucose; filled symbols, cells measured in the presence of glucose. Black circles, the wild-type strain; red triangles, Δ*ndhF1*; and blue squares, Δ*gnd*.

Thus, the later induction phase from the I-level at around 70 ms to the P-level may contain the information about the redox state of the PSI acceptor side in the dark, which is determined by the condition of respiration. In order to quantify the rate of induction during this phase, we defined “T_peak_” as the time to reach 93% of the P-level (see Materials and Methods). The T_peak_ in Δ*ndhF1* is about two-thirds of the value in the wild-type strain, while it is about 150% in Δ*gnd* (Table 1). Addition of glucose decreased the T_peak_ to approximately 60% of that without glucose in the wild-type strain, while the effect of glucose was minor in Δ*ndhF1* and Δ*gnd*. This T_peak_ showed a strong correlation with the increase in the NADPH fluorescence upon illumination, which is the index of the level of oxidized NADP^+^ in the dark (Figure 5; *R*^2^ = 0.952). The result strongly suggests that chlorophyll fluorescence induction can be used for the estimation of the redox state of NADPH in the dark.

**Table 1:** Time to reach 93% of the P-level in ms (T_peak_).

	No addition	+Glucose
WT	178 ± 25	112 ± 7^b^
Δ*ndhF1*	117 ± 2^a^	110 ± 6
Δ*gnd*	270 ± 8^a^	250 ± 6^a^^b^

a Significant difference (*p* < 0.05) between the wild-type strain (WT) and ΔndhF1 or Δgnd under the same condition.

b Significant difference (*p* < 0.05) between cells measured with no addition and those in the presence of glucose.

## Discussion

### Interaction Between Photosynthesis and Respiration *via* the Acceptor Side of PSI

It is well accepted that the several components of electron transport, e.g., the PQ pool, are shared by photosynthesis and respiration in cyanobacteria (Aoki and Katoh, 1982; Peschek and Schmetterer, 1982), resulting in the direct interaction between the two electron transport chains (Hirano et al., 1980; Mi et al., 1992). The PQ pool is in equilibrium with the electron acceptor Q_A_ in PSII, the redox sate of which is a primary factor to determine the yield of chlorophyll fluorescence (Krause and Weis, 1984). Thus, the yield and induction of chlorophyll fluorescence are sensitive to the condition of respiratory electron transport in cyanobacteria (Schreiber et al., 1995; Campbell et al., 1998). Furthermore, here we show that the redox state of NADPH in the dark affects induction of chlorophyll fluorescence in *Synechocystis* sp. PCC 6803. In this interaction as well, the direct factor that determines the yield of chlorophyll fluorescence must be the redox state of Q_A_ and the PQ pool, not that of NADPH. Apparently, when reduced NADPH is accumulated in the dark before the measurements of fluorescence induction, the electron transport to the downstream of PSI should be blocked, leading to the faster reduction of the PQ pool under illumination.

The influence of the downstream of PSI on the induction of chlorophyll fluorescence through the changes in the redox state of the PQ pool is well predictable at least in theory. Actually, there have been several observations related to this point. For example, when the Calvin cycle was inhibited by the addition of D, L-glyceraldehyde to spinach leaves or by lowering the ambient CO_2_ concentration of barley leaves, the induction kinetics of the chlorophyll fluorescence was modified in a timescale of a minute to several minutes (Walker, 1981; Walker et al., 1983). Since stromal NADPH content was reported to increase 1 min after onset of illumination and it takes more than 100 s to reach the maximum reduction (Lim et al., 2020), the changes in the rate of NADPH consumption at the Calvin cycle must be reflected in the induction of chlorophyll fluorescence in the timescale of minutes. Compared with these results, NADPH/NADP^+^ dependent changes in the peak position of the chlorophyll fluorescence induction observed here were in the time range of several hundred milliseconds (Figures 3, 4). The difference may simply reflect the fact that the position of NADPH in the electron transport chain is closer to PSII and more direct, compared with rather “remote” NADPH consumption in the Calvin cycle. Although photoactivation (i.e., redox control) of the Calvin cycle enzymes may partly contribute to relatively long timescale of its effects on the fluorescence induction, precise characterization is very difficult due to the large effect of state transition in this time range, especially in the case of cyanobacteria (Papageorgiou et al., 2007).

Electron acceptor pool size of PSI must be also influenced by the activity of ferredoxin-NADP^+^ reductase (FNR), which is known to be inactive in the dark and photoactivated under light (Arakaki et al., 1997), just in the case of some Calvin cycle enzymes. In the case of land plant leaves, it was proposed that the final acceptor of electron transport within first 1 s of fluorescence induction should be ferredoxin, since photoactivation of FNR takes 1–2 s after the onset of illumination (Schansker et al., 2005, 2006; Stirbet and Govindjee, 2011). In the case of cyanobacteria, however, NADPH fluorescence of cells dark-acclimated for 15 min increased upon illumination within 150 ms (Figure 2). Similarly, fast rise of NADPH fluorescence was observed for the cyanobacterial cells dark-acclimated for 6–10 min (Kauny and Sétif, 2014; Sétif et al., 2020). Thus, dark-acclimation of cyanobacterial cells does not inactivate FNR in *Synechocystis* sp. PCC 6803 at least if dark-acclimation time is up to 15 min. In the case of algae, it was reported that the fluorescence induction kinetics of intact chloroplast of *Bryopsis maxima* dark-acclimated for 2 min before the measurements was affected by the addition of an inhibitor of FNR, phenylmercuric acetate (Satoh and Katoh, 1981). In their study, addition of the inhibitor suppresses “dip,” a temporal decrease in fluorescence yield observed at 100 ms after the onset of illumination, suggesting that the electron transport through FNR could serve to oxidize the PQ pool under physiological conditions.

### Two Sites of Metabolic Interaction: The PQ Pool and Stroma

As discussed above, the redox state of NADPH should affect the induction of chlorophyll fluorescence through the acceptor side of PSI. In the case of cyanobacteria, however, the redox state of NADPH can be affected not only by photosynthetic electron transport or the Calvin cycle but also by respiratory electron transport. In both cases, the redox state of NADPH affects that of the PQ pool. However, the effect of the NADPH redox is reflected in the changes in the PQ redox only during illumination through photosynthetic electron transport, while under the dark-acclimated condition, it is through respiratory electron transport. In this study, we used two gene-disrupted mutants with different NADPH redox but with the same PQ pool redox under dark-acclimated condition, to elucidate the effect of NADPH on the PQ pool through photosynthetic electron transport.

We could demonstrate that the redox state of NADPH affected the peak position of chlorophyll fluorescence induction through photosynthetic electron transport. The comparison of two gene-disrupted mutants in itself, however, does not exclude the possibility that the redox state of the PQ pool determined during the dark-acclimation through respiratory electron transport also affects the fluorescence peak position. Nevertheless, we assume that the effect of the redox state of the PQ pool in the dark-acclimated cells on the peak position is rather minor based on the following observations. The fluorescence peak positions determined in the presence of glucose were very similar in the wild-type strain and in Δ*ndhF1* (Figures 3B, 4D). On the other hand, the PQ pool in the dark-acclimated cells in the presence of glucose should be reduced in the wild-type strain but oxidized in Δ*ndhF1*. Thus, the effect of the redox state of the PQ pool in the dark-acclimated cells on the peak position should be minor compared with the effect of the NADPH redox through photosynthetic electron transport. This is also consistent with our previous report that the faster rise to the peak in Kautsky curve in Δ*ndhF1* was hardly affected by the addition of KCN, which should fully reduce the PQ pool during dark-acclimation (Ogawa et al., 2013).

In our OJIP transient, the fluorescence level during the first 70 ms (corresponding to O-J-I phase) is high in the wild-type strain while it is low in the two gene-disrupted mutants (Figures 4A,B), in which the PQ pool is more oxidized after dark-acclimation (Ogawa et al., 2013; Figure 1). When the PQ pool was reduced by chlororespiration on the contrary, the fluorescence level rapidly increased to the maximum level at J-step (around 2 ms after illumination) in broken chloroplast in *Pisum sativum* (Haldimann and Strasser, 1999). Apparently, these results suggest that the more the PQ pool is reduced in the dark before the measurements, the higher the level of fluorescence at J-step is. The effect of the redox state of the PQ pool on the J-step may be independent of the redox state of NADPH in the dark, since the difference in the induction of chlorophyll fluorescence around the J-step is relatively small between Δ*ndhF1* and Δ*gnd* (Figures 4C,D), which should have totally different redox state of NADPH.

In addition to the J-level, the O-level can be considered to reflect the redox state of the PQ pool, which changes depending on the condition of respiration in cyanobacteria (Papageorgiou et al., 2007). When the PQ pool is reduced by the respiratory chain during dark-acclimation, the O-level would be high due to the partial suppression of photochemical quenching. The high fluorescence peak in Δ*ndhF1* and Δ*gnd* (Figure 3) can be explained by the following reason. Although the redox state of NADPH is totally different in these two disruptants, PQ must be more oxidized in both strain in the dark compared with the wild-type strain, which is reflected in the lower O-level, and thus gives higher peaks in the induction curves that is normalized at the O-level (Figure 3).

In summary, the redox state of NADPH is reflected in the dark-to-light induction of chlorophyll fluorescence separately from the effect through respiratory electron transport. In the OJIP transient, the redox state of the PQ pool in the dark-acclimated cells is reflected in O-J-I phase during the initial 70 ms of illumination, while the redox state of NADPH is reflected in the subsequent changes in I-P phase. In the Kautsky curve, the redox state of the PQ pool is reflected in the peak height relative to the O-level while the redox state of NADPH is reflected in the initial peak position.

### Assessment of the Redox State of the PSI Acceptor Side by the Dark-to-Light Induction Curve of Chlorophyll Fluorescence

We observed that the strong correlation exists between an index of the peak position of chlorophyll fluorescence induction (T_peak_) and the increase in the NADPH fluorescence upon illumination, which is the index of the level of oxidized NADP^+^ in the dark (Figure 5). Thus, the time to reach the peak level in the induction of chlorophyll fluorescence can be potentially used for an index of the redox state of the PSI acceptor side in the dark in cyanobacteria. Although the redox state of NADPH can be determined non-destructively by NADPH fluorescence in parallel with the measurements of PSII by chlorophyll fluorescence, the measurements of chlorophyll fluorescence are far easier compared with the measurements of NADPH fluorescence. Usually, fluorescence signal of NADPH is much smaller than that of chlorophyll. Furthermore, cyanobacterial cells often secrete substantial fluorescent materials to culture medium during growth, so that the baseline fluorescence signal is high even after washing of the cells with fresh culture medium. If the redox state of NADPH can be determined by conventional chlorophyll fluorometer, the estimation of the redox condition of the PSI acceptor side becomes much easier.

It must be noted, however, that the dark-to-light induction kinetics of chlorophyll fluorescence is affected not only by the redox state of NADPH but also by many factors. We showed that the redox state of the PQ pool in the dark-acclimated cells did not much interfere the estimation of the redox state of NADPH. However, chlorophyll concentration or cell density of the sample certainly interferes it. Since, the method is based on the fact that the fluorescence rise reflects the balance between electron’s flow-in to the PQ pool and flow-out from the PQ pool under illumination during the measurements. We observed that sample with higher concentration exhibited the slower induction of the chlorophyll fluorescence (data not shown). This can be attributed to the self-shading effect, which makes the rate of electron transport to the PQ pool slower by weakening actinic light. Actually, when we screened the data in the Fluorome, the cyanobacterial fluorescence kinetics database, for the gene-disrupted mutants with altered peak position, many of the candidates are slow-growth strain which showed faster rise to P-level because of the decreased self-shading. Nevertheless, we assume that the chlorophyll fluorescence measurements are useful for the initial screening of the mutants with altered redox state in the PSI acceptor side. Especially, when considering the fact that very few mutants should show higher growth rate than wild-type strain, delayed peak phenotype may be a good index for the oxidized NADPH in the dark. Although the peak position of the chlorophyll fluorescence induction may not be used for the precise quantification of the redox state of NADPH, it can be used for the initial screening for the mutants with altered redox state around the acceptor side of PSI by the simple analysis of OJIP curve or the Kautsky curve.

## Data Availability Statement

The original contributions presented in the study are included in the article/Supplementary Material, and further inquiries can be directed to the corresponding author.

## Author Contributions

KSo designed the study. TO and KSu performed the experiments. TO and KSo wrote the manuscript. All authors contributed to the article and approved the submitted version.

## Conflict of Interest

The authors declare that the research was conducted in the absence of any commercial or financial relationships that could be construed as a potential conflict of interest.

## Publisher’s Note

All claims expressed in this article are solely those of the authors and do not necessarily represent those of their affiliated organizations, or those of the publisher, the editors and the reviewers. Any product that may be evaluated in this article, or claim that may be made by its manufacturer, is not guaranteed or endorsed by the publisher.

## Footnotes

^1^ http://www.photosynthesis.jp/fluorome/
