# Supplementary material for: Respiration Interacts With Photosynthesis Through the Acceptor Side of Photosystem I, Reflected in the Dark-to-Light Induction Kinetics of Chlorophyll Fluorescence in the Cyanobacterium Synechocystis sp. PCC 6803
Source: Front Plant Sci. 2021 Jul 28;12:717968. doi: 10.3389/fpls.2021.717968 (PMC8355559; doi:10.3389/fpls.2021.717968)
Supplement: Supplementary file 1 [file Image_1.pdf]

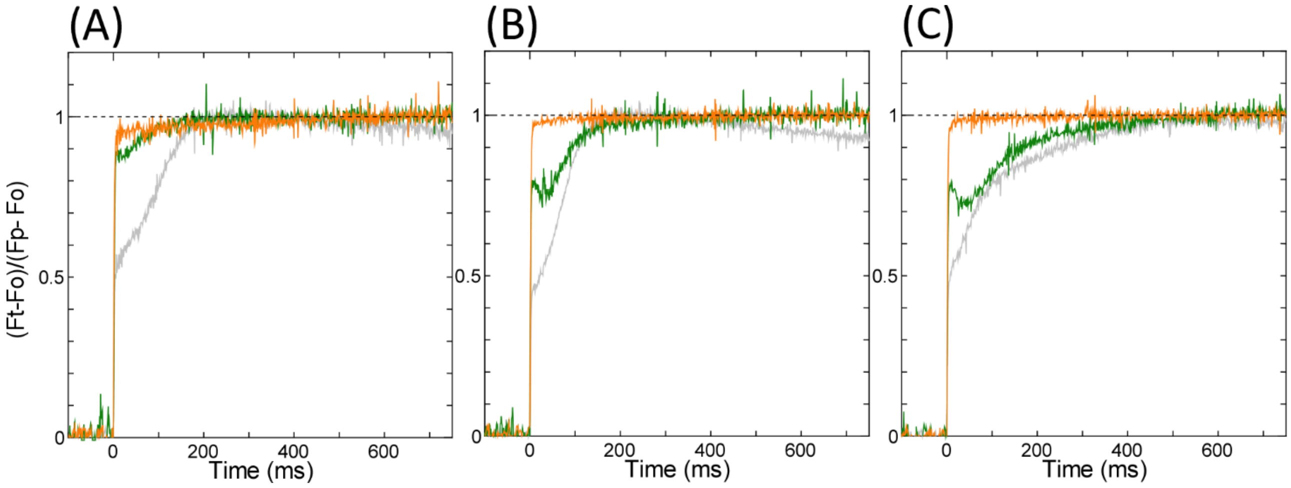

Supplementary Figure 1. The dark-to-light induction kinetics of chlorophyll fluorescence upon saturating pulse of 800 ms in the wild-type strain (A), Δ*ndhF1* (B) and Δ*gnd* (C). Gray line, without any addition; green line, in the presence of 20 μM DBMIB; orange line, in the presence of 10 μM DCMU. A black dashed line represents the level under the condition where Q_A is fully reduced. The average of three independent cultures is presented.
